# Intraguild Predation and Native Lady Beetle Decline

**DOI:** 10.1371/journal.pone.0023576

**Published:** 2011-09-13

**Authors:** Mary M. Gardiner, Matthew E. O'Neal, Douglas A. Landis

**Affiliations:** 1 Department of Entomology, Michigan State University, East Lansing, Michigan, United States of America; 2 Department of Entomology, Iowa State University, Ames, Iowa, United States of America; Duke University, United States of America

## Abstract

Coccinellid communities across North America have experienced significant changes in recent decades, with declines in several native species reported. One potential mechanism for these declines is interference competition via intraguild predation; specifically, increased predation of native coccinellid eggs and larvae following the introduction of exotic coccinellids. Our previous studies have shown that agricultural fields in Michigan support a higher diversity and abundance of exotic coccinellids than similar fields in Iowa, and that the landscape surrounding agricultural fields across the north central U.S. influences the abundance and activity of coccinellid species. The goal of this study was to quantify the amount of egg predation experienced by a native coccinellid within Michigan and Iowa soybean fields and explore the influence of local and large-scale landscape structure. Using the native lady beetle *Coleomegilla maculata* as a model, we found that sentinel egg masses were subject to intense predation within both Michigan and Iowa soybean fields, with 60.7% of egg masses attacked and 43.0% of available eggs consumed within 48 h. In Michigan, the exotic coccinellids *Coccinella septempunctata* and *Harmonia axyridis* were the most abundant predators found in soybean fields whereas in Iowa, native species including *C. maculata*, *Hippodamia parenthesis* and the soft-winged flower beetle *Collops nigriceps* dominated the predator community. Predator abundance was greater in soybean fields within diverse landscapes, yet variation in predator numbers did not influence the intensity of egg predation observed. In contrast, the strongest predictor of native coccinellid egg predation was the composition of edge habitats bordering specific fields. Field sites surrounded by semi-natural habitats including forests, restored prairies, old fields, and pasturelands experienced greater egg predation than fields surrounded by other croplands. This study shows that intraguild predation by both native and exotic predators may contribute to native coccinellid decline, and that landscape structure interacts with local predator communities to shape the specific outcomes of predator-predator interactions.

## Introduction

In many areas of the U.S., human-mediated disturbances have altered the landscape, resulting in a matrix of agricultural and urban land uses containing fragmented patches of semi-natural habitats. These landscapes support altered food webs which contain both accidently and intentionally introduced species at multiple trophic levels. The introduction of non-native species is considered a major threat facing native biodiversity [1]. Among introduced and native generalist predators, both direct and indirect competitive interactions can influence predator-prey dynamics and the stability of native predator populations [2], [3], [4]. Therefore, evaluating competitive interactions occurring between native and introduced species is critical to understanding potential threats to the stability of native predator biodiversity and biocontrol services.

An important example is the soybean-soybean aphid system. The soybean aphid *Aphis glycines* Matsumura, a native of Asia, was first detected in the U.S in July of 2000. Both the primary and secondary host plants of the aphid; common buckthorn, *Rhamnus cathartica* L. (Rhamnaceae) and the cultivated soybean, *Glycine max L.* (Fabaceae); as well as its complex of lady beetle predators, *Harmomia axyridis* Pallas, *Coccinella septumpuncata* L., *Hippodamia variegata* (Goeze), and *Propylea quatuordecimpunctata* (L.) (Coccinellidae) are all introduced species. Although the addition of these exotic coccinellids into U.S. agricultural food webs has contributed to the biocontrol of *A. glycines* and other aphid pests, increasing evidence suggests that their presence has also resulted in the displacement of native coccinellid competitors. In recent decades declines in several native coccinellids have been documented in the U.S., including *Coccinella novemnotata* Herbst, *Coccinella transversoguttata richardsoni* Brown, *Adalia bipunctata* (L.), *Brachiacantha ursina* (F.), *Cycloneda munda* (Say), *Chilocorus stigma* (Say), and *Hippodamia convergens* Guérin-Méneville [5], [6], [7], [8], [9], [10], [11]. The decline of these species has been dramatic, for example *C. novemnotata* was once a common agricultural species and is now quite rare [10].

Although exotic coccinellids have been implicated as the cause of native decline, potential competitive mechanism(s) are not fully understood. One hypothesis is that native coccinellid decline is due to enhanced interference competition via intraguild egg and larval predation (IGP). Exotic coccinellids have been observed to act as intraguild predators of native coccinellids in laboratory and field cage studies [12], [13], [14], [15], [16], [17], however, the extent to which IGP via egg predation occurs within agroecosystems has not been previously studied.

In addition, recent studies have illustrated that agricultural landscape structure may influence coccinellid communities and therefore the intensity of potential competitive interactions such as IGP. Gardiner et al. [18] measured native and exotic coccinellid diversity and abundance in soybean fields across Iowa, Michigan, Minnesota, and Wisconsin in 2005–06 and found that the proportion of the coccinellid community composed of native species varied significantly across this region, from a low of 10% natives in Michigan to 44.8% natives in Iowa. They found that the abundance of native and exotic lady beetles was tied to the composition of the landscape surrounding soybean fields. The presence of semi-natural habitat within the agricultural landscape was important for both native and exotic species; however, the type of habitat present influenced the community structure. Landscapes with an abundance of forested habitat had the greatest proportion of the *H. axyridis* while landscapes with an abundance of grasslands supported higher populations of native species.

This study investigated the extent of egg predation experienced by a native coccinellid, *Coleomegilla maculata* (De Geer) in soybean fields. The goal of this research was to determine if egg predation could be a significant factor influencing changes in the native coccinellid community. Our specific objectives were to: 1) Determine if native coccinellid egg predation occurred within soybean fields, 2) Examine whether egg predation was correlated with the abundance of exotic coccinellids present, and 3) Determine if egg predation was influenced by local, edge, or large-scale landscape composition. Our initial hypothesis was that landscapes with an abundance of forested habitat (i.e. Michigan), known to support higher exotic coccinellid populations, would support greater egg predation of a native coccinellid than landscapes which limit the success of exotic Coccinellidae (i.e. Iowa).

## Methods

### Selection of sentinel species

We selected *Colelomegilla maculata* as a model to measure the amount of egg predation experienced by native coccinellids in the agricultural landscape. This species is a common native coccinellid throughout the Eastern U.S. and is found in soybean in both Iowa and Michigan [18], [19], [20], [21]. Females were easily collected and readily produced egg masses in culture, and thus, could be used as a sentinel.

### Egg mass collection

Beginning in May 2007, *C. maculata* adults were collected from old field grasslands and alfalfa fields near the Iowa State University campus (Ames, IA) using sweep nets. Females were placed individually into Petri dishes with strips of paper to serve as an oviposition substrate. Beetles were provided daily with water, honey, and eggs of the corn earworm, *Helicoverpa zea* (Boddie). Dishes were checked daily for *C. maculata* eggs and any found were frozen (−80°C). For transport from IA to MI, egg masses were packed in dry ice and transported by van from Iowa State University to Michigan State University where they were stored in a −80°C freezer until they were deployed in the field. We determined that 15 eggs per oviposition event were the average number of eggs deposited by *C. maculata* under these conditions and therefore we used this size egg mass in our field experiment. To prepare sentinel egg masses, individual eggs were either added or removed from existing masses using a paint brush, to reach the desired number of eggs per strip (n = 15). Added eggs were attached to the paper strip using water soluble glue (Elmer's Products, Columbus, OH). The standardized egg masses were cut out and glued onto filter paper disks (12 mm diameter).

### Measuring predation of frozen egg masses

To determine if freezing *C. maculata* eggs affected predation, we compared consumption of previously frozen and live *C. maculata* eggs by four predators commonly found in soybean fields: *C. septempunctata*, *H. axyridis*, *H. parenthesis* and *Nabis* sp. Individual predators were released into a Petri dish arena containing three thawed and three fresh *C. maculata* eggs, which were randomly assigned to 2×3 grid. Predation of the fresh and thawed eggs was measured at 8, 18, and 24 h. Ten replicates were completed for each predator species. A mixed effects repeated measures analysis of variance (ANOVA) model was used to determine if predators were as likely to consume fresh versus frozen eggs. Fixed factors included in the model were egg treatment (fresh or frozen), time (8, 18, or 24 hr) and a treatment by time interaction.

### Field sites

During July of 2007, we measured the intensity of predation on sentinel egg masses of *C. maculata* in soybean fields in Michigan (n = 8) and Iowa (n = 6). A minimum distance of 10 km separated each field site. Field size averaged 15.1 ha (range: 3.0–60.4 ha). Within each state, soybean fields were located within landscapes which ranged from agriculturally-dominated to diverse landscapes containing both agricultural and semi-natural habitats. Within each soybean field, all sampling took place within four 0.2 ha plots established at least 30 m from any edge. Soybean fields were not treated with insecticide during the study period.

### 48 h egg predation field experiment

To distinguish egg predation from other forms of loss (desiccation, physical dislodgement etc.), predator accessible and exclusion treatments were compared. The exclusion treatment consisted of one egg mass enclosed in a 22 cm cage to prevent predators from accessing the eggs. The predator accessible egg treatment consisted of an un-caged egg mass. A total of 8 egg masses were present per field site, (one of each treatment within each of the four plots). To begin the experiment, two filter paper disks containing egg masses were glued onto the top side of a soybean trifoliate leaf approximately 15 cm from ground level on adjacent plants in the center of each plot. Both treatments remained in the field for 48 h after which they were collected and examined to determine the number of undamaged eggs remaining. After 48 h of exposure in the field 40% of eggs in the caged treatment were partially collapsed, mimicking damage that could be caused by piercing sucking predators. To avoid attributing damage which may have been caused by freezing and thawing to predation, we only considered eggs to be damaged if there was clear evidence of attack by a chewing predator. Thus our study does not account for losses due to piercing-sucking predators and as such, is likely to be a conservative estimate of *C. maculata* egg predation.

### Nocturnal egg predation experiment

To account for the proportion of the egg predation due to nocturnally active predators, we conducted a predation experiment at night in eight sites (four per state). The two egg mass treatments (predator accessible and exclusion) were placed within the center of each plot on adjacent plants at dusk and removed at dawn (9 h exposure from 2000–2200 h to 0500–0700 h) and the number of undamaged eggs recorded.

### Soybean aphid and predator survey

During the 48 h experiment, we measured the activity of potential egg predators by two methods; yellow sticky traps and sweep netting, previously shown effective in describing the natural enemy community in soybeans [22]. We placed one unbaited yellow sticky card (PHEROCON AM, Great Lakes IPM, Vestaburg, MI) in the center of each plot. A metal “T” fence post was erected and a 22.9×27.9 cm sticky card was suspended just above the plant canopy. Sticky traps remained in the field for 48 h. All predators were counted and identified to species. We also measured the abundance of predators by collecting a 20-sweep sample from two rows of soybean plants in each plot. As the abundance of extraguild prey may affect the intensity of egg predation, we also measured soybean aphid populations within each field site using destructive plant counts. In each plot, five randomly selected plants were removed from the ground and the number of apterous and alate *A. glycines* were counted on each plant.

### Landscape analysis

Field geospatial data were collected using a handheld GPS receiver using Wide Area Augmentation System (WAAS) correction. The spatial coordinate (WSG 1984) for the center of each field was used to obtain ortho-rectified digital aerial imagery for the site. We digitized the habitats surrounding each study site to a radius of 2 km using ARC GIS 9.0 and conducted ground verification of each landscape from July–August 2007. Each landscape polygon within 2 km of the center of the study sites was given a value corresponding to one of seven landscape categories: corn, soybean, other crops, forest, grassland, wetland, or urban. Some locations included polygons that were not visible from a roadway and permission to access private lands could not be obtained. These polygons were given a value of zero and were excluded from further analysis. However, the area of each site that could not be identified was very low (<1%). The smallest polygons identified included field plots on university research farms and small patches of abandoned crop field (<5 m^2^) the largest were contiguous forests, grasslands and crop fields (≤1.3 km^2^).

Using ARC GIS 9.0 we measured characteristics of the landscape surrounding each field site. The composition of the habitat edge surrounding each soybean field site was quantified by determining the proportion of the perimeter bordered by cropland edge, semi-natural edge or urban edge. Semi-natural edge included all grasslands and forested habitats. Urban edges included residential land and roadways. We determined landscape heterogeneity within a 2 km radius from the center of each field using Simpson's Index (D) [23]. Simpson's Index is typically used to examine the variance of species abundance distributions; here we applied it to examine variance in the proportion of area covered by each of seven land cover categories. The equation for Simpson's Index (D) is: D = 1/Σ(p_i_)^2^ where p_i_ = proportion of habitat in the i^th^ land-cover category (D increases as landscape heterogeneity increases).

### Statistical analysis

Both field experiments (48 h and nocturnal) tested two null hypotheses. The first was that within a field, predators are not a significant source of egg injury (no difference in number of damage eggs in exclusion versus predator accessible cages). In our model this factor is designated as Treatment. Our second null hypothesis was that sentinel egg predation does not vary by State. As described earlier, the ratio of exotic to native coccinellids in the soybean fields of MI and IA vary significantly. In this way, State is a proxy within our statistical model for landscapes that vary in their coccinellid communities. To avoid confounding land-use within the treatment factor, we selected locations in each state so that the range of agricultural land use was similar. To test both null hypotheses, a split-plot mixed effects analysis of variance model (ANOVA) was used. This model included Treatment (Exclusion and Predator Accessible egg masses) and State (Michigan and Iowa) as fixed effects and a State by Treatment interaction. Random effects were Site (multiple field locations within each state) nested within State and Plot nested within Site and State.

A log likelihood chi-square test assuming a multinomial distribution was used to examine the variation in the community of potential egg predators in Michigan and Iowa soybean fields [24]. This test determines if the species composition of the predator community within each state was significantly different. The null hypothesis for this test was that the proportional distribution of predator species within Michigan and Iowa did not differ. This test was completed using compiled species data from sticky cards and sweep net samples. Both the ANOVA models and log likelihood chi-square test were completed using SAS v. 9.1 [25].

To evaluate the relationship between native coccinellid egg predation and landscape variables, we performed a principal components analysis (PCA) to reduce the dimensions of the data. Six landscape variables (Forest, Grassland, Corn, Soybean, Other Crops, and Urban) and three edge variables (Semi-natural Edge, Cropland Edge, and Urban Edge) were included in the PCA analysis. To meet the assumption of a multivariate normal distribution of the variables, the landscape variable Wetland was dropped prior to analysis as it made up a very small proportion of the 14 landscapes (average of 1.4%, range of 0–8.2%). Principal component axes were extracted using correlations among variables and the resulting factors were not rotated [26]. We restricted our analysis to the first three eigenvectors which explained 73.8% of the variability in the data.

To assess the influence of within-field, and landscape variables on the abundance of egg predators and the intensity of native coccinellid egg predation, multiple models were compared using Akaike's Information Criterion, adjusted for a small sample size (AIC_c_) [27]. The abundance of potential egg predators was estimated by summing the mean number of predators collected per sweep sample and yellow sticky card trap for each site. This combined mean was log (x+1) transformed prior to analysis to meet the assumptions of normality and homogeneity of variances (SAS Institute, 1999). The mean number of *C. maculata* eggs remaining after 48 h was also log (x+1) transformed prior to analysis. The relationship between the response variable Eggs Remaining (number of eggs remaining in the predator accessible treatment after 48 h of exposure) and nine models were examined (Table 1). Relationships between the response variables Potential Egg Predators and Potential Exotic Egg Predators and seven models were compared (Table 1). We selected the model with the minimum AIC_c_ value as having the best support for the data, and considered any model with an AIC_c_ difference of less than two from the best fit model to be a competing model [27], [28]. For each model, we present the maximum log-likelihood estimate, the Akaike weights, which estimate the relative likelihood of a given model against all other models, and AIC_c_ differences (Δ_i_). We calculated adjusted r^2^ to evaluate how well the models explained the variation in the data. The AIC_c_ analysis and adjusted r^2^ were determined using R version 2.1.1 [29].

**Table 1:** Models compared using Akaike's Information Criterion, adjusted for a small sample size (AIC_c_) for the response variable Eggs Remaining (number of eggs remaining in the predator accessible treatment after 48 h of exposure).

Model	Explanation of Variable
**Area**	Area of soybean fields where experiments were conducted
**Perimeter**	Perimeter of soybean fields where experiments were conducted
**Prey**	Average abundance of soybean aphid present within each site
**Potential Predators^1^**	Average abundance of all potential egg predators collected in sweep samples+average abundance of all potential egg predators collected on yellow sticky card traps
**Potential Exotic Predators^2^**	Average abundance of exotic potential egg predators collected in sweep samples+average abundance of exotic potential egg predators collected on yellow sticky card traps
**D**	Simpson's Index of landscape heterogeneity, calculated at a radius of 2 km surrounding the study sites
**PC1**	Principal component 1 interpreted from Principal Components Analysis
**PC2**	Principal component 2 interpreted from Principal Components Analysis
**PC3**	Principal component 3 interpreted from Principal Components Analysis

For the analysis of Eggs Remaining the variables (1) Potential Predators and (2) Potential Exotic Predators were included as predictors. These were also examined as response variables and a total of seven models were examined (Area, Perimeter, Prey, D, PC1, PC2, and PC3).

## Results

### Egg predation

In the laboratory, all four predators tested (*H. axyridis*, *C. septempunctata*, *H. parenthesis*, and *Nabis* sp.) consumed an equivalent number of frozen and live eggs (the variable Treatment was not significant at *P*<0.05). For *H. parenthesis*, *C. septempunctata*, and *Nabis* sp. egg predation increased over time, resulting in a significant time effect.

**Figure 1:**
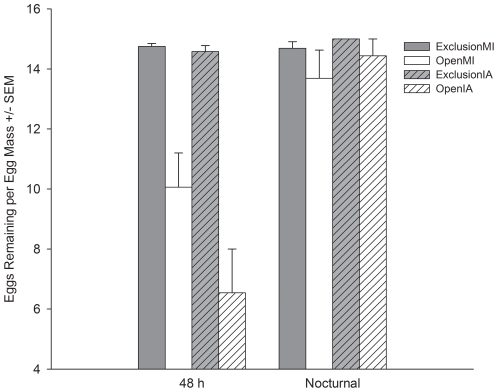
Mean number of eggs remaining in the predator exclusion cage and predator accessible treatments in Iowa and Michigan soybean fields for the 48 h and nocturnal (9 h) predation experiments.

In the field, egg masses of *C. maculata* were subject to significant predation in soybean fields across Michigan and Iowa after 48 h of field exposure (F_1,54_ = 45.7, *P*<0.001) (Figure 1). In the predator accessible treatment, 60.7% of egg masses were attacked by predators and 43.0% of available *C. maculata* eggs were consumed after 48 h of exposure. Across all egg masses in the predator accessible treatment, 8.6±0.9 eggs remained of the original 15 per mass after 48 h. There was a marginally significant difference in the amount of predation incurred between the states (State*Treatment interaction: F_1,54_ = 3.2, *P* = 0.0804), with *C. maculata* eggs in Iowa soybean experiencing slightly higher predation than in Michigan fields (6.5±1.5 and 10.1±1.1 eggs remaining in predator accessible egg masses in Iowa and Michigan respectively).

### Nocturnal egg predation experiment

In the nocturnal predation test there was no significant difference between the number of eggs remaining in predator exclusion and predator accessible treatments (F_1,30_ = 2.0, *P* = 0.1725) (Figure 1). Across the states, an average of 14.1±0.5 eggs remained of the original 15 per egg mass in the predator accessible treatment.

### Aphid and predator populations

Aphid populations in soybean fields during the egg experiment were low across the 14 sites, varying from 0 (Monroe and Britton, MI) to 21.4±4.5 per plant (Ames, IA). In both states low numbers of chewing predators were detected, averaging 0.3±0.1 and 0.7±0.1 per 20 sweeps in Iowa and Michigan respectively (Table 2). Chewing predators on yellow sticky cards averaged 0 per card in Iowa and 1.6±0.2 in Michigan. Six species of potential egg predators were found: *H. axyridis*, *C. septempunctata*, *P. quatuordecimpunctata*, *C. maculata*, *H. parenthesis* and the soft-winged flower beetle *Collops nigriceps* (Say) (Melyridae) (Table 2). The species composition of these communities in Michigan and Iowa soybean fields were significantly different (X^2^_5_ = 28.3, P<0.0001). In Michigan, the community was dominated by the coccinellids *H. axyridis* and *C. septempunctata*. In Iowa, *C. nigriceps* comprised 50% of the predator community followed by *C. maculata*, *H. parenthesis* and *H. axyridis* which each accounted for 16.7%. As C. *nigriceps* was not included in preliminary testing, we subsequently measured its potential as a predator of *C. maculata*. Four specimens were placed into individual Petri dishes each with a thawed *C. maculata* egg mass. Within 3 h, three of four *C. nigriceps* consumed all 15 eggs; in the fourth replicate the beetle did not consume any eggs after 24 h.

**Table 2:** Percent of the total predator community and mean abundance ± SEM of predators found in Iowa and Michigan soybean fields.

	Sweep Net^a^			
	Percentage of Total		Mean ± SEM	
Predator Species	Iowa	Michigan	Iowa	Michigan
*H. axyridis*	16.7	33.3	0.04±0.04	0.22±0.07
*C. septempunctata*	0.0	57.1	0	0.38±0.11
*P. quatuordecimpunctata*	0.0	4.8	0	0.03±0.03
*C. maculata*	16.7	0.0	0.04±0.04	0
*H. parenthesis*	16.7	4.8	0.04±0.04	0.03
*C. nigriceps*	50.0	0.0	0.13±0.09	0
**Total**	100	100	0.25±0.12	0.66±0.12

	Yellow Sticky Card Trap^b^			
	Percentage of Total		Mean ± SEM	
	Iowa	Michigan	Iowa	Michigan
*H. axyridis*	0	27.0	0	0.31±0.09
*C. septempunctata*	0	70.3	0	0.81±0.20
*H. parenthesis*	0	2.7	0	0.03±0.03
**Total**	0	100	0	1.16±0.20

a Sweep samples consisted of a 20-sweep sample of two rows of soybean plants.

b Yellow sticky cards were placed just above the plant canopy and remained in the field for 48 h (coincident with egg predation experiment).

### Edge and landscape composition

The composition of the habitat edge surrounding the 14 study sites ranged from 0 to 81.4% and 0 to 37.8% semi-natural habitats in Iowa and Michigan respectively. Cropland borders ranged from 0 to 70.4% (Iowa) and 28.6 to 76.6% (Michigan) whereas urban habitats comprised 0 to 34.4% (Iowa) and 0 to 63.0% (Michigan) of field site edges.

Within a 2 km landscape radius surrounding each of the 14 sites, landscape diversity values (Simpson's D) ranged from 2.1 to 5.3. The percentage of the landscape composed of cropland ranged from 18.1 to 94.9%. Landscapes with high and low percentages of these cropland occurred in both Michigan (18.1 to 94.9%) and Iowa (23.2 to 87.8%). Grassland habitat comprised 7.2 to 64.9% of Iowa and 0 to 25.9% of Michigan landscapes. Forested habitat comprised from 0.04 to 21.5% of Iowa and 1.2 to 21.5% of Michigan landscapes.

### Interpretation of principal components

**Figure 2:**
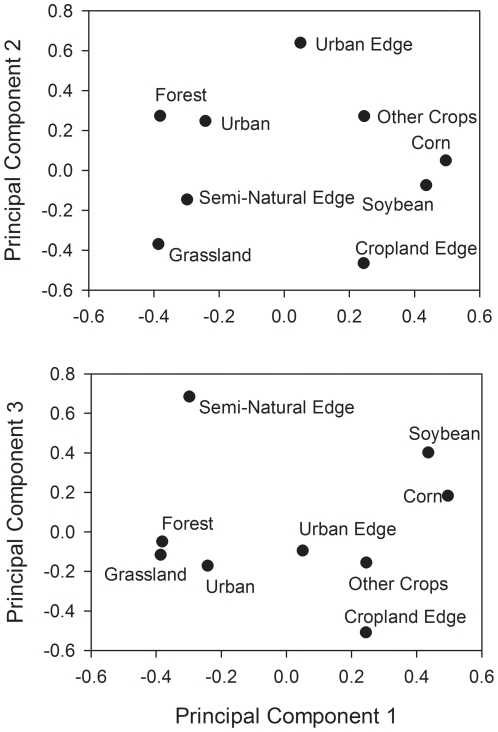
PCA ordination for principal components (PC) 1–3 landscape variables sampled at a radius of 2 km and edge variables bordering soybean fields. Points indicate the principal component loadings of each variable included in the PCA analysis. Sites with positive loadings on PC1 were correlated with the variables Corn and Soybean while negatives loadings on PC1 were correlated with the variables Forest and Grassland. Sites with positive loadings on PC2 were correlated with the variable Urban Edge while negative loadings on PC2 were correlated with the variables Cropland Edge. Sites with positive loadings on PC3 were correlated with the variable Semi-Natural Edge while negative loadings on PC3 were correlated with the variable Cropland Edge.

The first principal component (PC1) was a measure of landscape composition. Positive loadings on PC1 were correlated with the variables Corn and Soybean while negative values were correlated with the variables Grassland and Forest (Figure 2). Therefore sites with positive values of PC1 suggest a landscape with an abundance of corn and soybean agriculture whereas sites with negative values of PC1 indicate a landscape with a diversity of semi-natural habitats. Both PC2 and PC3 were related to the composition of the edge immediately surrounding soybean field sites. For PC2, sites with positive loadings were correlated with the variable Urban Edge and negative loadings were correlated with the variable Cropland Edge. Edges of sites with low values of PC2 were composed primarily by cropland while the edge of sites with high values of PC2 included roadways and residential habitat. For PC3, sites with positive loadings were correlated with the variable Semi-Natural Edge and negative loadings were correlated with the variable Cropland Edge. Agricultural lands dominated the habitats bordering field sites with low values of PC3 whereas semi-natural habitats such as forests and grasslands dominated the edges of field sites with high values of PC3 (Figure 2).

### AIC_c_ analysis of native coccinellid egg predation and predator abundance

**Figure 3:**
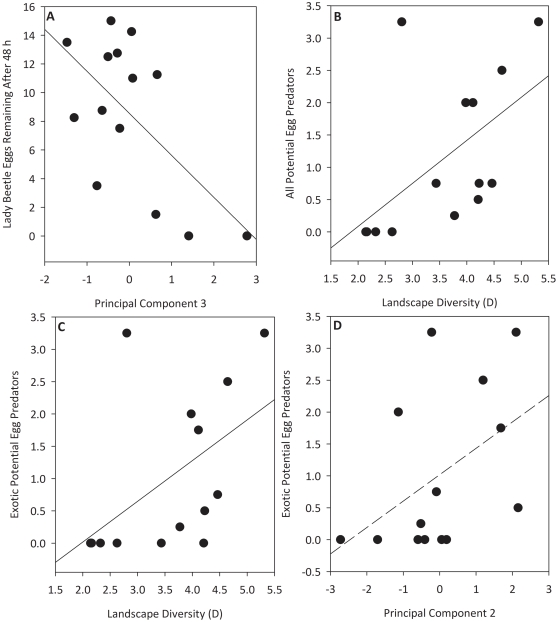
Relationships between principal components, egg predation and predator abundance. (A) Negative relationship between PC 3 (a measure of edge composition) and the number of *C. maculata* eggs remaining after 48 h of exposure to predators in soybean fields (*P* = 0.002). Soybean fields boarded primarily by semi-natural habitats had high values of PC3 whereas soybean fields bordered by cropland had low values. (B) Positive relationship between landscape diversity (Simpson's D) and the abundance of potential lady beetle egg predators (*P* = 0.012). Diverse landscapes supplied a larger number of predators to soybean fields compared with simple landscapes dominated by cropland. (C) Positive relationship between landscape diversity (Simpson's D) and the abundance of potential exotic lady beetle egg predators (*P* = 0.034). Diverse landscapes supplied a larger number of exotic predators to soybean fields compared with simple landscapes dominated by cropland. (D) Relationship between PC2 and the abundance of potential exotic lady beetle egg predators (*P* = 0.071). Soybean fields boarded primarily by urban habitats had high values of PC2 whereas soybean fields bordered by cropland had low values. Egg and predator data was log (x+1) transformed prior to analysis, untransformed means are shown here for interpretation.

The PC3 model predicting the abundance of eggs remaining after 48 h had the lowest AIC_c_ value (Table 3). There was a significant (*P* = 0.002) negative relationship between PC3 and the number of eggs remaining in the open egg treatment after 48 h (Figure 3). This indicates that the intensity of native coccinellid egg predation in soybean fields was greater when fields were bordered by semi-natural habitats rather than agricultural fields. None of the other models examined qualified as a competing model (Δ_i_<2 of best fit model) (Table 3). For the response variable Potential Egg Predators, the landscape diversity (D) model had the lowest AIC_c_ value of all candidate models examined, no competing models were found for this response variable (Table 3). Potential Exotic Egg Predators was also best predicted by the landscape diversity (D) model. For this response variable, the PC2 model was a competing model (Table 3). There were significant positive relationships between landscape diversity and the abundance of potential egg predators (*P* = 0.012) and potential exotic egg predators (*P* = 0.034). This illustrates that diverse landscapes, which in this study contained both natural and agricultural land supplied a larger predator community to soybean fields compared with simple landscapes dominated by corn and soybean. There was a marginally significant relationship (*P* = 0.076) between PC2 and exotic predator abundance. This indicates that a weak positive relationship exists between the proportion of the soybean field surrounded by urban land use and potential exotic egg predator abundance.

**Table 3:** Summary of AIC_c_ model selection statistics for evaluating (1) the intensity of *C. maculata* egg predation in soybean fields in Iowa and Michigan, (2) the abundance of all potential egg predators and (3) the abundance of exotic potential egg predators.

Response	Model^1^^,^ 2	Log-likelihood	K_i_	AIC_c_	Δ_i_	W_i_	Adjusted r^2^
Eggs Remaining	**y = ** ***B_o_*** **+** ***B_1_*** (PC3)***	**−13.15**	**3**	**34.70**	**0.00**	**0.92**	**0.53**
	y = *B_o_*+*B_1_*(Area)*	−17.16	3	42.72	8.02	0.02	0.17
	y = *B_o_*	−18.99	2	43.06	8.36	0.01	
	y = *B_o_*+*B_1_*Exotic Predators	−17.46	3	43.32	8.62	0.01	0.13
	y = *B_o_*+*B_1_*Perimeter	−17.6	3	43.60	8.90	0.01	0.11
	y = *B_o_*+*B_1_*All Predators	−17.68	3	43.76	9.06	0.01	0.10
	y = *B_o_*+*B_1_*D	−18.12	3	44.64	9.94	0.01	0.04
	y = *B_o_*+*B_1_*P(PC1)	−18.82	3	46.04	11.34	0.00	−0.06
	y = *B_o_*+*B_1_*(PC2)	−18.96	3	46.32	11.62	0.00	−0.08
	y = *B_o_*+*B_1_*(Prey)	−18.99	3	46.38	11.68	0.00	−0.07
Potential Predators	**y = ** ***B_o_*** **+** ***B_1_*** D*	**−7.19**	**3**	**22.77**	**0.00**	**0.71**	**0.38**
	y = *B_o_*+*B_1_*PC2	−9.11	3	26.62	3.85	0.10	0.17
	y = *B_o_*	−11.06	2	27.19	4.42	0.08	−0.05
	y = *B_o_*+*B_1_*(PC3)	−10.28	3	28.96	6.19	0.03	0.03
	y = *B_o_*+*B_1_*(Perimeter)	−10.74	3	29.88	7.11	0.02	−0.04
	y = *B_o_*+*B_1_*(Area)	−10.86	3	30.11	7.34	0.02	−0.06
	y = *B_o_*+*B_1_*(Prey)	−10.90	3	30.20	7.43	0.02	0.17
	y = *B_o_*+*B_1_*(PC1)	−11.06	3	30.51	7.74	0.01	−0.80
Potential Exotic Predators	**y = ** ***B_o_*** **+** ***B_1_*** D*	**−9.26**	**3**	**26.93**	**0.00**	**0.41**	**0.27**
	**y = ** ***B_o_*** **+** ***B_1_*** PC2*	**−10.02**	**3**	**28.44**	**1.51**	**0.19**	**0.18**
	y = *B_o_*+*B_1_*(PC3)*	−10.32	3	29.05	2.12	0.14	0.15
	y = *B_o_*	−12.01	2	29.09	2.16	0.14	
	y = *B_o_*+*B_1_*(Perimeter)	−11.83	3	32.07	5.14	0.03	−0.06
	y = *B_o_*+*B_1_*PC1	−11.88	3	32.16	5.23	0.03	−0.06
	y = *B_o_*+*B_1_*(Prey)	−11.88	3	32.16	5.23	0.03	−0.06
	y = *B_o_*+*B_1_*(Area)	−11.97	3	32.34	5.41	0.03	−0.08

The minimum AIC_c_ model for each response variable and any competing models (Δ_i_<2) are shown in bold.

1 Variables in parentheses indicate a negative relationship with response variable.

2 * *P*<0.1, *** *P*<0.01.

## Discussion

This study measured the intensity of native coccinellid egg predation experienced by *C. maculata* within soybean fields, a habitat utilized by both native and exotic coccinellid species [18], [30], [31]. Our goals were to determine the percentage of *C. maculata* eggs removed by predators, the timing of predation (noctural or diurnal) and whether predator community composition or presence of extraguild prey influenced egg predation. Furthermore, we determined how various landscape features (area and perimeter of soybean fields, structure of edge habitats, large-scale landscape heterogeneity, and landscape composition) influenced egg removal by predators.

### Egg predation of *C. maculata*

Eggs of *C. maculata* were readily consumed by predators within soybean fields. To accurately identify predation pressure on a specific prey, it is critical to account for activity of both diurnal and nocturnal natural enemy species [32]. Although we did not detect a significant difference in the number of eggs remaining in the predator accessible and exclusion treatments in the nocturnal study, the number of eggs missing from the open treatment was consistent, on a per hour basis, to the 48 hr study. If egg predation is assumed to occur at constant rate across the 48 h experiment, predators removed 0.9% of available egg masses per hour. The rate of egg removal during the nocturnal predation study is 0.7% per hour. Therefore, the influence of nocturnally active predators clearly should not be ignored. Pfannenstiel and Yeargan [32] observed that *C. maculata* larvae, Phalangiidae, *Clubiona abbotii* (Clubionidae), Elateridae, Carabidae, and *Lygus lineolaris* (Lygeidae) were all nocturnally active predators of lepidoptern eggs in corn and soybean fields. As we use the predator data collected from sweep and yellow sticky card sampling methods to explain the variation in egg predation, it should be noted that some predators may have been missed by these methods which may be important contributors to egg predation.

### The influence of potential egg predators

A total of six species of predators known to consume coccinellid eggs were collected via sweep net and yellow sticky trap sampling. These included three exotic coccinellids (*C. septempunctata*, *H. axyridis*, and *P. quatuordecimpunctata*), two native coccinellids (*C. maculata* and *H. parenthesis*), and the melryid *C. nigriceps*. Prior to this study, predation of coccinellid eggs by *C. nigriceps* had not been reported in the literature. While the overall extent of egg predation did not vary between Michigan and Iowa, the species collected from study fields varied. Exotic species occupied a larger percentage of the coccinellid community in Michigan versus Iowa. In the exotic-dominated food webs of Michigan, predation of native coccinellid eggs may be contributing to the maintenance of exotic-dominated populations. In Iowa, native species made up a greater proportion of the predators, and native coccinellid eggs support both native and exotic species.

Interestingly, we found that neither the abundance of all potential predators (six species) or the abundance of exotic coccinellids alone (three species) were strong predictors of egg predation. Therefore, our hypothesis that soybean fields with a greater number of exotic coccinellids experience higher levels of egg predation was not supported. This suggests that while exotic lady beetle species may have contributed to overall predation of this native species, they do not unilaterally drive the result. As we did not attempt to observe predation events in the field, we do not know the precise identity of the predators which attacked *C. maculata* eggs. However, based on the guild of predators sampled from our field sites we hypothesize that a complex of both native and exotic predators contributed to predation of this species. Still, future work is needed to quantify the role of IGP by specific predators in shaping native coccinellid communities.

### Influence of landscape composition on predators egg predation

Large-scale landscape structure was an important predictor of predator abundance but not a of egg removal. Instead, we found that the structure of local edge habitats influenced the intensity of *C. maculata* egg predation in soybean fields. Fewer *C. maculata* eggs remained after 48 h of exposure to predation within soybean fields bordered by semi-natural habitats than in soybean fields boarded by cropland. The composition of semi-natural habitat edges was diverse, including pasturelands (36.9%), forests (31.1%), forage (23.5%), old field (5.9%) and restored prairie (2.5%). As we found no relationship between the abundance of the egg predators sampled and the amount of predation detected, it is likely that additional species were supplied by these edge habitats which also fed upon *C. maculata*. These may include other arthropods (spiders, opiliones, ants, carabid beetles) and potentially birds or rodents not currently known to feed on coccinellid eggs and thus, not accounted for by the sampling methods we employed. One possibility is that while the predators we anticipated respond to landscape structure at larger landscape scales (i.e. the response of coccinellids to landscape diversity and overall composition) that these lesser known predators are responding at finer grain scales (i.e. to specific edge habitats). Indeed, many of these potential predators are inherently less mobile or have edge-oriented behaviors which may focus their abundance and impacts along edges. Alternatively, because we utilized freeze-killed eggs, a portion of the egg removal we observed may have been due to the activity of scavengers versus true predators that may also have differing habitat requirements and movement patterns. Future studies examining the entire egg predator community found in field edges and their impacts on native coccinellid eggs will be required to provide mechanistic explanations. In addition, techniques that allow discerning scavenging from predation may be particularly useful [33].

### Summary, Implications, and Future Work

This study demonstrates that eggs of the native coccinellid, *C. maculata* within soybean fields are subject to intense predation from a variety of native and exotic species. Our original hypothesis was that soybean fields within landscapes which supply the largest populations of exotic coccinellids would experience the highest predation of *C. maculata* eggs, however, we found nearly equal IGP in landscapes dominated by native and exotic predators. In contrast to overall landscape structure driving exotic predator abundance and impact, we found the composition of the habitats immediately bordering soybean fields was the strongest predictor of egg losses. Soybean fields surrounded by semi-natural edges including habitats such as forests, restored prairies, old fields and pasturelands experienced greater egg predation than fields surrounded by other croplands and multiple intraguild predators, both native and exotic, may contribute to native coccinellid decline.

A caveat is that the factors we observed influencing predation on *C. maculata* may or may not be the same as on other native coccinellids. Future research should also examine the role of IGP by both native and exotic predators on rare or declining native coccinellids such as *C. novemnotata*, *C. transversoguttata richardsoni*, *A. bipunctata*, and *H. convergens*. Moreover, future studies should investigate the interactions of local predator communities and landscape structure in shaping the specific outcomes of predator-predator interactions. However, because landscapes with semi-natural field edges also promote increased pest control [34], [35], [36] our results suggest that it may be difficult to simultaneously manage landscapes to promote pest suppression without also increasing IGP on native coccinellids.
